# A novel arginine regulated PhD pathway

**DOI:** 10.1038/s41467-023-44473-8

**Published:** 2024-01-03

## Abstract

As part of our tenth anniversary celebrations, the editorial team at *Nature Communications* wanted to hear from early career researchers who have published with us. We asked the early career researchers to tell us in an essay what is amazing about the research question(s) that drove them and the highs—and lows—of the journey from hypothesis to publication.

**Figure:**
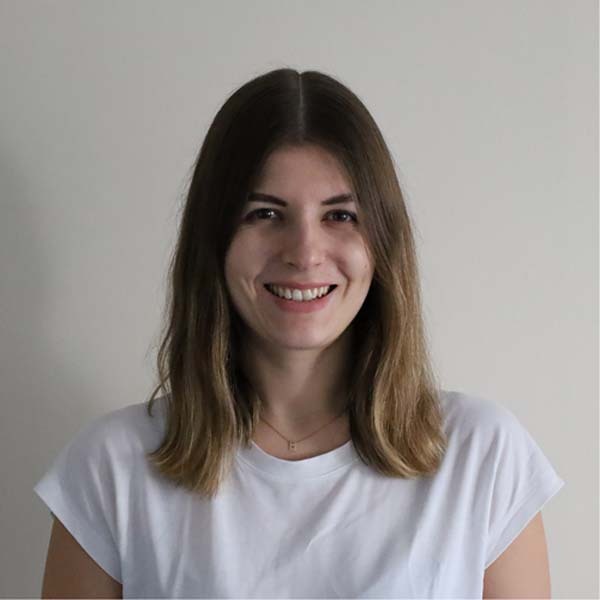
Julia Brunner

Julia Brunner’s scientific career started in Gernot Schabbauer’s lab at the Medical University Vienna. After finishing her Master’s at CeMM in Vienna, she returned to the Schabbauer Lab for her PhD. There, she published her paper on the role of arginine in osteoclasts formation. Julia’s general interests revolve around (immuno)metabolism, cell biology and particularly in cellular and metabolic cross-talk in organ niches. Julia finished her PhD at the end of April 2020 (which was not easy, considering the year!) and is currently doing a postdoc in New York at the Memorial Sloan Kettering Cancer Center in Lydia Finley’s lab.

Q: As an early career researcher and an author of a paper published by *Nature Communications*^1^, tell us about the journey you’ve been on.

**A:** So imagine, you just finished your Master’s degree. You are hopeful, fresh and motivated, and you are excited and amazed because you will actually be paid to pursue your interests. You think you know exactly how academia works and are slightly annoyed by the fact that the only advice people give you is to choose your mentor and project wisely. Maybe you are adventurous and decide to change countries and topic, eager to invest, not only in your education, but also in your personal development. Maybe you’d quite like to stay in the city you’ve been calling home (I mean, they do not call Vienna the most livable city in the world for nothing) and in the lab of the young PI who first showed you how to properly hold a mouse when you just turned 20. Indeed, like many other scientific career pathways, mine is a story of successes and failures, breakdowns and breakthroughs, Reviewer 2 and Reviewer 3. But let’s start at the beginning.

When I first started my PhD I got assigned a main or “safer” project (with preliminary data to build upon) and a side or “risky” project (no data yet, exciting hypothesis). The side project was introduced to me as follows: “We got this drug from a collaboration partner. I think it might do something very interesting to your cells!”. Little did I know that this was my first encounter with the enzyme that would bring tremendous joy and simultaneously haunt me for the next 5 years: arginase 1. Arginase 1 is a crucial player of the urea cycle and degrades the amino acid arginine into urea and ornithine. Why did I allow arginase 1 to take center stage in my scientific endeavors? In contrast to other metabolic genes, whose expression is tightly controlled, arginase 1 can be highly induced and released by macrophages to limit arginine availability, thereby it’s a potent dampener of adaptive immune responses. Indeed, the importance of arginine for T cells has been extensively shown, but as a proud member of team “innate immunology,” my culture was not a T cell one. I was culturing osteoclasts, which are gigantic, multinucleated, bone resorbing macrophages. In the dish, my readout was simple: starting with equal amounts of wildtype or genetically modified bone marrow precursor cells, adding in different cellular cues and ultimately counting osteoclast numbers after a fixed number of days. If lucky, I would usually observe differences that resulted in a quarter more or less formed osteoclasts. After the addition of arginase to my culture, something striking happened: I did not observe a single osteoclast. At first, I thought that this was a rookie mistake—probably the enzyme concentration was too high. However, titrating the enzyme revealed that my cells were “fine”, they just kept looking like happy precursor cells, sitting in the dish, awaiting arginine’s return, enabling them to fulfill their destiny to become beautiful giant cells. Did you by now already forget that I had a “safety project”? Yeah, me too. “How does the restriction of extracellular arginine by arginase 1 completely halt osteoclast formation?” was the question I now mumbled to myself before falling asleep, ready to dream about a metabolite that could substitute for arginine absence (spoiler: still didn’t find one). After securing that treatment with my favorite enzyme exerted beneficial effects in three different models of murine arthritis, I embarked on a mission to unravel the mechanisms. This journey resulted in the detailed collection of a gigabyte of negative data. I am still not exactly sure how arginine starvation blocks osteoclast formation, but I extensively published on the osteoclast-specific pathways that aren’t mediating it!

Fast forward four years, 3 months and 19 days, there it was—a finished manuscript. The process of “writing up” was not easy. Collaborators received a constant flow of e-mails, figure 1 turned to figure 3 and then ended up as figure 2, my 2nd author, a computational biologist, was confronted with a lot of last-minute data analysis. I had the feeling there were still experiments that I wanted to do, but I felt an enormous feeling of accomplishment, pride and excitement. After I hit the send button, the submission process started, and well, it was a tiring one. I was not aware that at prestigious journals the editorial decision to even send out or reject papers can take up to a month or longer, as countless papers are submitted every day. Anyhow, with every day passing, my hopes grew bigger, pushing away the fear of rejection. Maybe they liked it, right? Maybe they just have to ask for the opinion of an advisor and you know, people are busy! But after writing a reminder e-mail, the waiting ended and rejection finally rolled over me, washing away my big, unrealistic dreams for where I would publish my story (ever heard about journal IF?). However, there was a silver lining: an editor at *Nature Communications* agreed to directly send it out to reviewers, if only we agreed! This was a huge deal for me, knowing my work will be finally evaluated and judged by valued members of the scientific community. Three reviewers were assigned and after a time span that again felt like forever, I got back positive feedback, but major revisions. Some comments were harsh, but mostly reasonable and doable and I was ready to give it my all. After my PI had a nice conversation with the editor, it felt like we knew which experiments to focus on, so I went on and did them. We revised the manuscript, implemented the new data and wrote up a rebuttal, and were confident and thankful—after all, revisions in general significantly improve a manuscript! We were very positive, thinking our paper found a home and would be accepted for publication. Unfortunately, I would have never expected the reaction of Reviewer 2 (of whom I had only heard of in the realms of twitter) as he/she was very unhappy with the new data, especially with experiments that I performed to answer Reviewer 3’s comments. I was frankly quite devastated. After talking to our assigned editor, we agreed on doing an extensive literature search with the goal to thoroughly address all concerns raised. After confronting Reviewer 2 with our opinion, he/she was not satisfied and was now saying that our manuscript should be rejected, around 8 months after I first hit the submission button. What to do now? Clearly in distress, we contacted our editor again, who agreed to send it to Reviewer 3 to make the final call. My mind was blank—should I start reformatting? Have I just lost almost a year of my life? On top of that, my PhD program requires a first authorship to graduate. Will I ever finish? Days went by, until just before Christmas Eve we got the reassuring news from Reviewer 3 and the acceptance letter for our paper (Brunner et al. *Nat. Commun.* **11**, 431 (2020)). What a relief—it was the best Xmas present ever!

So what did I learn from this process? Rest assured, I still love science and will continue facing acceptances and rejections and the connected highs and lows as a postdoc. I try to tell myself that reviewers are just scientists, some with opinions diverging from your own, and that’s OK. Also, there is life apart from work and although the downs of the publishing process are an inherent part of the scientific system, they do not tell anything about the valuable aspects of being a scientist. Ultimately, the data is the data! During the revision process, I also performed experiments showing that multinucleated giant cells, which are similar but different to osteoclasts, behaved comparably to my bone eating friends as soon as their arginine supply was removed. Therefore, the reviewer comments even led to the opening up of new research avenues in our lab, with PhD students continuing to work on preliminary data that I generated. Even after I’m gone, my research will be continued—Thanks, *Nature Communications* for making this possible and for publishing my first paper!
